# Setting research priorities to achieve long-term national road safety goals in Iran

**DOI:** 10.7189/jogh.12.09002

**Published:** 2022-04-02

**Authors:** Homayoun Sadeghi-Bazargani, Alireza Razzaghi, Ali Atabak, Shahrzad Bazargani-Hejazi, Shahriar Behzad Basirat, Leila Doshmangir, Salman Ebrahiminejad, Mostafa Farahbakhsh, Rahim Farahnak Benekohal, Saiedeh Ghaffarifar, Mina Golestani, Mohammad Hossein Hamidi, Seyed Taghi Heydari, Einollah Jahani, Leila Jahangiry, Ali Imani, Mohammad Mehdi Khabiri, Abolfazl Khishdari, Hamed Marouf, Gholamreza Masoumi, Adel Mazloumi, Mohammad Reza Mehmandar, Seyed Abdolreza Mortazavi-Tabatabaei, Khalil Pourebrahim, Nasir Baradaran Rahmanian, Forouzan Rezapur-Shahkolai, Mahdi Rezaei, Mohammad Saadati, Ehsan Sarbazi, Ezat Samadipour, Mojtaba Sehat, Mehdi Shafieian, Afshin Shariat Mohaymany, Hamid Soori, Saeedeh Sheikhi, Masoud Tabibi, Jafar Sadegh Tabrizi, Ali Tavakoli Kashani, Ibrahim Vahabzadeh, Salah Veisi, Mirbahador Yazdani

**Affiliations:** 1 Road Traffic Injury Research Center, Tabriz University of Medical Sciences, Tabriz, Iran; 2 Managing Director of Rahpooyan Consulting Engineers, Tehran, Iran; 3 Department Psychiatry, College of Medicine, Charles R. Drew University of Medicine and Science and UCLA David Geffen School of Medicine, Los Angeles, California, USA; 4 Strategic Crisis Management, Research Institute for Law Enforcement and Social Studies, NAJA, Tehran, Iran,; 5 Department of Health Policy & Management Research Center, School of Management and Medical Informatics, Tabriz, Iran; 6 Social Determinants of Health Research Center, Tabriz University of Medical Sciences, Tabriz University of medical Sciences, Tabriz, Iran; 7 Social Determinants of Health Research Center, Tabriz University of medical Sciences, Tabriz, Iran; 8 Vehicle Dynamical Systems Research Laboratory, School of Automotive Engineering, Iran University of Science and Engineering, Tehran, Iran; 9 Research Center of Psychiatry and Behavioral Sciences, Tabriz University of Medical Sciences, Tabriz, Iran; 10 Civil and Environmental Engineering, University of Illinois Urbana-Champaign, Newmark Civil Engineering Building, Urbana, Illinois, USA; 11 Medical Education Research Center, Health Management and Safety Promotion Research Institute, Tabriz University of Medical Sciences, Tabriz, Iran; 12 The Police Force of the Islamic Republic of Iran, Tehran, Iran; 13 Health Policy Research Center, Institute of Health, Shiraz University of Medical Sciences, Shiraz, Iran; 14 Amin Police University, Tehran, Iran; 15 Health Education and Health Promotion Department, School of Public Health, Tabriz University of Medical Sciences, Tabriz, Iran; 16 Health Economics Department, Tabriz Health Service Management Research Center, Tabriz University of Medical Sciences, Tabriz, Iran; 17 Department of Civil Engineering, School of Engineering and Technology, Yazd University, Yazd, Iran; 18 Department of Civil Engineering, Yazd University, Yazd, Iran; 19 Road Maintenance and Transportation Organization, North Khorasan, Iran; 20 Emergency Management Research Center, Iran University of Medical Sciences, Tehran, Iran; 21 Department of Occupational Health Engineering, School of Public Health, Tehran University of Medical Sciences, Tehran, Iran; 22 Faculty member of Amin (NAJA) University, Tehran, Iran; 23 Proteomics Research Center, Shahid Beheshti University of Medical Sciences, Tehran, Iran; 24 Traffic Safety Department, General Directorate of Highways, Khorasan Razavi, Iran; 25 Department of Public Health, School of Public Health & Social Determinants of Health Research Center, Hamadan University of Medical Sciences, Hamadan, Iran; 26 Khoy University of Medical Sciences, Khoy, Iran; 27 Department of Operating Room and Anesthesia, School of Paramedic Sciences, Sabzevar University of Medical Sciences, Khorasan Razavi, Iran; 28 Department of Biosciences and Epidemiology, Kashan University of Medical Sciences, Kashan, Iran; 29 Amirkabir University of Technology (Tehran Polytechnic), Department of Biomedical Engineering, Tehran, Iran; 30 Iran University of Science and Technology, Tehran, Iran; 31 Safety Promotion and Injury Prevention Research Center, School of Public Health, Shahid Beheshti University of Medical Sciences, Tehran, Iran; 32 Tabriz Health Services Management Research Center, Iranian Center of Excellence in Health Management, Tabriz University of Medical Sciences, Tabriz, Iran; 33 School of Civil Engineering, Iran University of Science and Technology, Tehran, Iran- Road safety research center, Iran University of Science and Technology, Tehran, Iran; 34 Ministry of Roads and Transportation, Road Safety Commission, Tehran, Iran; 35 Department of Architecture, Faculty of Art and Architecture, University of Kurdistan, Sanandaj, Iran

## Abstract

**Background:**

Road traffic crashes (RTCs) and its associated injuries are one of the most important public health problems in the world. In Iran, RTCs rank second in terms of mortality. To address this issue, there is a need for research-based interventions. Prioritizing researches using a variety of approaches and frameworks to determine the most effective interventions is a key nodal point in the RTCs' research policy planning cycle. Thus, this study aims to generate and prioritize research questions in the field of RTCs in Iran.

**Methods:**

By adapting the Child Health and Nutrition Research Initiative (CHNRI) method, this study engaged 25 prominent Iranian academic leaders having role in setting Iran’s long-term road safety goals, a group of research funders, and policymakers. The experts' proposed research questions were independently scored on a set of criteria: feasibility, impact on health, impact on the economy, capacity building, and equity. Following the prioritization of Research Questions (RQs), they were all classified using the 5 Pillar frameworks.

**Results:**

In total, 145 Research Questions were systematically scored by experts against five criteria. Iran's top 20 road traffic safety priorities were established. The RQs related to “road safety management” and “road and infrastructure” achieved a high frequency.

**Conclusions:**

The top 20 research questions in the area of RTCs in Iran were determined by experts. The majority of these RQs were related to “road safety management”. The results of this study may contribute to the optimal use of resources in achieving long-term goals in the prevention and control of road traffic crashes and its related injuries. Considering these RQs as research investment options will improve the current status of Road Traffic Injuries (RTIs) at a national level and further advance toward compliance with international goals. If these research priorities are addressed, and their findings are implemented, we can anticipate a significant reduction in the number of crashes, injuries, and deaths.

Road traffic injuries and deaths are a major public health problem, especially in low-and middle-income countries (LMICs). LMICs account for more than 90%of all crash fatalities worldwide. Among LMICs, Iran has one of the highest mortality rates from road traffic injuries and deaths. According to the 2018 Global Status Report on Road Safety, the estimated rate of RTI deaths in Iran was 20.5 per 100 000 people [1]. Road Traffic Injuries (RTIs) are the third leading cause of death worldwide but significantly higher in Iran, where they are the second leading cause of death behind cardiovascular diseases (CVDs) [2].

Iran has made significant progress in combatting various infectious and non-infectious disease but no significant progress has been made in the fight against RTIs due to on any effective interventions [3,4]. Despite significant progress in health, road traffic crashes and their associated injuries and deaths are one of the most serious public health problems in Iran. Numerous interventions have been implemented over the years; however, there have been no significant reductions in the incidence of crashes and related injuries. [2].

Preventing RTIs and deaths require special efforts because patterns vary between high, middle, and low-income countries. However, some countermeasures are globally applicable while others require further research and innovation [5,6]. Low and middle-income countries (LMICs) suffer from limited research resources and budgets [7]. Moreover, evidences leading road safety management is limited in LMICs and needs extensive researches [8]. Therefore, prioritizing research questions (RQs) is critical for research management in these countries [9]. In the past, many of efforts in road safety had no sufficient effectiveness to reduce road traffic crashes and the resulting deaths for various reasons. Several reasons including poor evidence-based decision making, lack of involvement of experts in various relevant fields, and lack of reliance on scientific evidence, are the main challenges in road safety management beside the other reasons [10]. However, scientifically based road safety management strategies are required to reduce crashes and related injuries [11]. Findings of different studies show that research in the field of health system is not consistent with the burden of disease or injuries and national health requirements [9,12]. The absence of research priorities can result in a waste of financial resources and time [9]. In 2018, a study was conducted to determine Iranian health system’s research priorities. Research in the field of RTIs was suggested as one of the top 10 priorities of the Iranian health system [12,13]. This demonstrates the significance of RTCs' challenges among health experts and stakeholders. Due to limited financial resources and budgets for RTIs, setting the priorities for RQs can help avoid excess costs and time and ensure that the allocated budgets are used effectively.

Prioritizing research questions can be accomplished by different methods. The Child Health and Nutrition Research Initiative (CHNRI) is one of the methods for prioritizing RQs. In 2005, the CHNRI was introduced by the World Bank with the aim of introducing a method to determine and identify research priorities in the health system. The method is based on the collective optimism of a group of experts [14]. It is a systematic and transparent prioritization process in health research, which involves researchers, policy makers, and stakeholders [14,15]. The CHNRI method has been used in many cases by national and international organizations [16,17]. Its utilization in prioritizing RQs in RTIs can be an efficient way for the country's health system and partner organizations to prevent RTCs and RTIs.

Overall, because of high rate of RTCs, their related injuries, and fatalities, it is critical to pay close attention to this issue. On the other hand, due to several significant challenges including a lack of evidence-based decision making, a lack of involvement of experts in various relevant fields, and limited research resources and budgets it is necessary to prioritize the RQs based on the opinions of experts in various related fields.

This study aims to use the CHNRI method to prioritize RQs in the area of RTCs in Iran. The findings may result in the most effective allocation of resources toward long-term goals of preventing and responding RTCs and their associated injuries.

## METHODS

The current study is a cross-sectional study that was conducted in Iran in 2020. In this study, the CHNRI method was used to prioritize RQs in RTCs. In this study engaged 25 prominent Iranian academic leaders having role in setting Iran’s long-term road safety goals, a group of research funders, and policy makers. The experts' proposed research questions were independently scored using a set of criteria including: feasibility, impact on health, impact on economy, capacity building, and equity. The method provides the possibility for professionals to independently generate research questions and limit the impact of group members on others [15]. Following that, experts provide comments, the score for each RQ is calculated, and the strengths, weaknesses, and rank of each RQ are determined based on their assessment [15]. Additionally, the method's systematic nature, transparency, reproducibility, and exclusive criteria to obtain information are several of its known advantages [14-16].

According to the purpose, in current study, RTCs experts in the country were identified and after achieving consent to participate, they were asked to give their RQs that could help the country in achieving long-term goals. After summarizing RQs, they were asked to rate them. Next to the scoring, 20 research priorities of the country were extracted to be considered for the next 5 years.

Iranian RTCs experts were identified from administrators and policymakers, police, health, roads and infrastructure, vehicles, behavior and culture, and academic researchers. Their identification was based on the following criteria: 1) systematic search for Iranian experts who were active in or outside the country in specialized fields related to RTCs in international bibliometric databases such as Scopus and Web of Science: and 2) detection by purposeful and later snowball sampling method. The research team members conducted a systematic search of published and gray literatures to find the documents related to road safety. Experts were found from the identified articles who were researcher in the field of road safety in Iran. The identified researchers were invited via email or phone call. All experts were initially informed that the study consisted of two stages: proposing the RQs followed by scoring them. Each expert was asked to indicate their willingness to participate in the study during each phase. Most of the experts participating in the study engaged in both phases. Using the snowball method, several renowned scholars and specialists in the field were asked to introduce other specialists who have held scientific roles in universities or scientific societies in Iran. Snowball sampling is one of the most popular methods of sampling in qualitative research to gain access to hard-to-reach populations [18]. At first, a number of initial famous experts (seeds) who met the research criteria were invited to participate in study. They were then asked to suggest additional specialists who met the research criteria. We used the social networks of initial famous experts to extend the participants’ chain. Finally, 25 experts expressed an interest in participating in the study. All experts who participated in the study were involved in both the generation and scoring of RQs. The steering committee approved the final list of expert names and ensured that all specialties were covered. Table S1 in the **Online Supplementary Document** contains information about experts who participated in this study.

The development of RQs was based on macro policies aimed at preventing RTCs. For this purpose, the steering committee began by compiling macro national and international policies on RTI prevention. These policies included relevant law in the National Development Plan, the National Road Safety Plan (NRSP) [17], and international goals for road safety such as the Sustainable Development Goals (SDGs) [19].

These policies and study instructions were emailed to each expert and they were asked to propose 3 to 5 RQs. The steering committee then reviewed them, and duplicates were eliminated, while some similarities were merged. Then, the research questions and scoring instructions were re-sent to the experts who were asked to rate each RQ based on the 5 criteria of feasibility, effect on health, effect on the economy, capacity building and equity.

In CHNRI, there are four answering options: 0, 0.5, 1 and no answer if the expert does not have enough information. However, the steering committee decided to update the scoring in the following: 3 for “yes”, 2 for “informed but undecided”, 1 for “no” and 0 for “insufficiently informed” to facilitate rating by respondents. Participants were assured that each anonymous expert would provide suggestions for RQs. **Table 1** contains the scoring criteria and definitions for research questions.

**Table 1:** Research Questions (RQ) scoring criteria and definitions

Number	Criterion	Definition
**1**	Feasibility	1) There is sufficient capacity (eg, information infrastructure) to conduct this research; 2) It is possible to provide training skills to the people who are going to do this research; 3) This research can be done while observing ethical considerations and it is possible to do all or part of it in the next five years and reach useful findings.
**2**	Impact on health	The findings of this study have a high potential to improve health through the following: 1) Reduce the incidence or prevalence of crashes; 2) Reduction of individual, social and environmental risk factors; 3) The effect on future planning in the field of road safety and the effect on the implementation of programs; 4) Improving the provision of health services by improving service acceptance, accessibility, appropriateness, effectiveness, efficiency and productivity, or making treatment methods or services less complicated; 5) Improving readiness of community and system to respond to health challenges
**3**	Impact on economy	The findings have a high potential to lead to the following: 1) The effect on the production of technology, products or services to the customer in direct way; 2) Optimization of previous products or services (including increasing quality or reducing production costs); 3) Knowledge-based entrepreneurship; 4) reducing the number of working days lost due to injury or disability of the injuries; 5) reducing the cost-opportunity of traffic crash victims or caregivers; 6) Reducing the direct costs of the injured patients in the health and welfare system; 7) Reducing the burden of indirect costs
**4**	Capacity building	The findings have a high potential to lead into the following: 1) Training human resources in Iran; 2) Creating new skills in the research team; Or 3) Investing in upgrading research facilities and equipment where the study will take place, such as traffic laboratory equipment
**5**	Equity	The findings have a high potential to lead into the following: 1) Interventions or services that are available to all people including vulnerable groups, and if it takes cost, would be affordable for everyone 2) Policies, programs, interventions or services that reduce inequality in RTIs. This goal can be achieved through policies or interventions that empower vulnerable groups to be less exposed to the crashes and its risk factors, or improve access to interventions or services.

The weighted research priority scores were used to rank RQs. Weighted RPSs were calculated following CHNRI guidelines. Weights were assigned to each criterion by dividing the average expected score by the mean assigned [20]. The weighted RPS was calculated using the following formula:

wRPS = [Feasibility ×0.77) + (Impact on Health × 0.88) + (Impact on Economy × 0.97) + (Capacity Building × 0.79) + (Equity × 0.86)/5]

Each question received an average score between of 0 and 1. The score for each question was then multiplied by 100 and ranged from 0 to 100. Additionally, the Average Expert Agreement (AEA) index was calculated for each question to determine the level of agreement among the scorers. The AEA was calculated by dividing the frequency of the mode (ie, the most common score) by the total number of scores.

Following the prioritization of RQ, the first 20 priorities were classified using the 5 pillars framework [21]. The 5 pillars framework was used to classify research priorities which can also determine how research questions are distributed in road safety. According to this framework, activities can be classified as belonging to one of the following pillars: road safety management, safe roads, safe vehicles, safe road users, or post-crash response [22]. The analysis in this study was conducted using Microsoft Office Excel 2016 (Microsoft Inc., Seattle WA, USA).

**Ethical approval and consent to participate**: This study approved by National center for Strategic Research in medical education with the research number of 961137.

## RESULTS

In total, 153 RQs were collected and scored systemically by 25 experts using five criteria. The gathered RQs were compared and contrasted for duplication and similarity. There was no duplication identified in suggested RQs. Only, a few suggested RQs were merged. Finally, 145 RQs were finalized. The top 20 road safety priorities for Iran are listed in **Table 2**. Table S2 in the **Online Supplementary Document** contains detailed information on all 145 RPS. The detailed information about all of 145 RPS is provided in Table S1 in **Online Supplementary Document**.

**Table 2:** The top 20 priorities for road traffic safety research in Iran

Equity (Score)	Capacity building (Score)	Impact on economy (Score)	Impact on health (Score)	Feasibility (Score)	AEA (%)	AEA (Average)	wRPS (Score)	RPS (Score)	Proposed RQ	Rank
57	69	65	70	66	76	19	55.8	65.4	Investigating the effects and strategies of developing intelligent systems to achieve safety goals in the country	1
60	63	66	70	64	76	19	55.2	64.6	Exploring ways to achieve public participation in promoting traffic safety	2
60	68	59	65	70	76	19	54.7	64.4	The investigation of methods for utilizing the experience and knowledge of experts and educated individual in the field of traffic safety	3
56	65	57	68	70	68	17	53.7	63.2	The study and compilation of significant RTC analysis programs by specialized crash analysis teams in conjunction with an in-depth examination of the crash scene in order to determine the causes of crashes and their severity	4
63	58	60	69	66	68	17	53.9	63.2	Study to assess and continuously improve the quality of RTCs-related health care services	5
49	63	66	65	72	68	17	53.7	63	Conducting pre and post intervention studies to ascertain the efficacy of road safety interventions	6
58	52	68	67	70	72	18	53.9	63	Evaluation of low-cost methods for improving road safety	7
57	58	61	67	71	68	17	53.5	62.8	Examining the possibility of amending road traffic-related legislation in order to improve road traffic safety	8
55	58	68	64	63	72	18	51.9	62	Reforming macro-level traffic safety policies	9
57	60	57	63	70	64	16	52.7	61.6	Study of the role of enforcing police regulations using modern and intelligent tools promote road traffic safety	10
54	64	58	62	68	68	17	49.1	61.4	Study of reviewing and developing a comprehensive RTCs registration system and analyzing indicators	11
63	57	56	65	64	68	17	52	61.2	Study of the challenges related to improving the safety of interurban and rural roads	12
54	57	63	66	65	64	16	52	61	Preliminary feasibility studies for establishing a vehicle rating and reporting system (accreditation) with the assistance of vehicle insurance companies	13
53	64	61	66	60	64	16	52.1	61	Study of the Lead Agency in road traffic crashes in Iran	14
54	58	58	66	68	68	17	47.5	61	Reviewing the road traffic rules related to driving violations in order to promote road safety and driving culture	15
60	60	57	64	62	68	17	51.9	60.8	What strategies are in place to develop continuous training of safe traffic behaviors for professional drivers (public transport drivers including buses, trucks, vans, and taxis)?	16
52	62	61	63	65	60	15	42.4	60.8	Study of mechanisms for boosting knowledge and industry exchange in the field of traffic safety	17
51	59	58	70	65	68	17	51.6	60.6	Study the possibility of an intelligent system for detecting driver fatigue and drowsiness	18
56	59	62	62	62	68	17	51.6	60.6	Study of establishing a connection between national intelligent systems such as the meteorological system, the police system, and the breakdown monitoring system on improving road traffic safety	19
60	55	66	55	64	64	16	51.6	60.6	Methodological study the process of attracting, providing, and allocating financial resources for road traffic infrastructure development in order to achieve sustainable development in the field of road traffic safety	20

RQ – Research Question, RPS – Research Priority Score, wRPS – weighted RPS, AEA – Average Expert Agreement

The level of agreement experts was between 37%-67%. The RQs with the highest level of expert agreement were generally those with the highest RPS.

The range for all 145 research questions was 36.6- 65.4. **Table 2** summarizes the top 20 research questions. The top priority RQ “Investigating the effects and strategies for developing intelligent systems to achieve safety goals in the country”, was identified to earn highest for impact on health and capacity building. Additionally, the second RQ dealt with road user behavior (RPS = 64.6), while the third RQ dealt with road safety management (RPS = 64.4).

The sixth priority with the RPS 63, would provide a high score for feasibility (feasibility score = 72). This RQ focused on conducting before-after studies to determine the effectiveness of road safety interventions.

The seventh priority “Evaluation of low-cost methods for improving road safety”, received the highest score for “impact on economy” (score = 68).

Both the fifth and twelfth priorities received the highest “Equity” score (score = 63). The fifth priority focused on improving the quality of health care services provided to road traffic accident victims, while the twelfth priority examined the challenges associated with improving the safety of interurban and rural roads.

The level of agreement between experts was between 60% and 72%. The RQs for with highest level of expert agreement were typically those with the highest RPS. The first, second, and third priorities had an AEA of 76%. The first was to “Investigating the effects and strategies of developing intelligent systems to achieve safety goals in the country”. The second priority was “Exploring ways to increase public participation in promoting traffic safety”. The third priority addressed “The investigation of methods for utilizing the experience and knowledge of experts and educated individual in the field of traffic safety”. The seventeenth priority had the lowest AEA at 60%. It was titled “Study of mechanisms for boosting knowledge and industry exchange in the field of traffic safety.” The weighted Research Priority Score (wRPS) for twenty priorities have been ranged from 55.8 to 42.2 (**Table 1**). Among the 145 RPS, the range of wRPS was 55.8 to 31.2.

**Figure 1:**
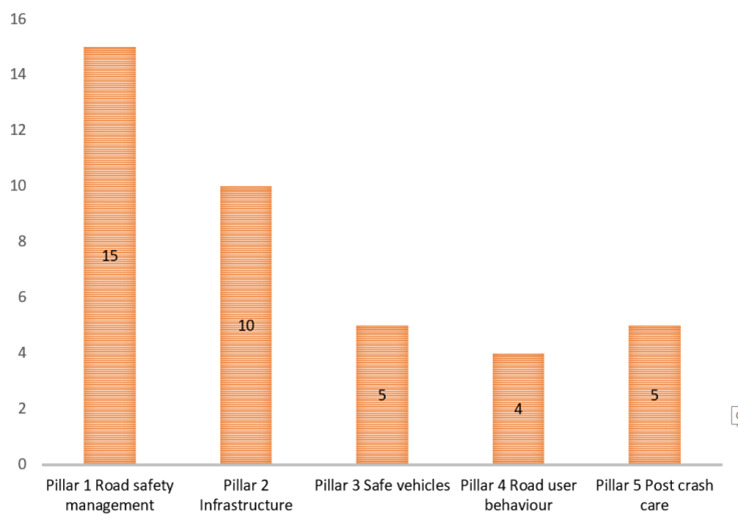
Distribution of the 20 identified priorities according to road safety pillars.

Then, the RQs were prioritized using the 5 pillars framework for road safety. **Figure 1** depicts the distribution of RQs in RTCs. The figure demonstrates that RQs associated with pillar 1 “road safety management” is the most frequently occurring. The second highest frequency was for road-and infrastructure-related RQs. The least frequency encountered RQs were those to the pillar of “road user safety.”

**Figure 2:**
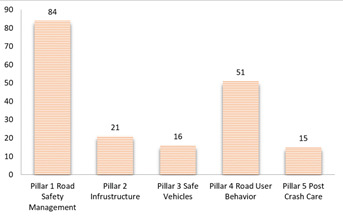
Distribution of all 145 priorities under Iran's road safety pillars.

Moreover, 145 RQs were classified using the 5 pillars framework for road safety. **Figure 2** depicts the distribution of all RQs. As illustrated in **Figure 1**, RQs related to pillar 1 “road safety management” is the most frequently occurring. The second frequent occurrence is associated with “road user behavior.” The RQs associated with the post-crash care pillar of has the lowest overall frequency in the total distribution.

## DISCUSSION

To our knowledge, this is the first time in Iran that a national-level road traffic research priority has been stablished for road traffic safety. The research questions in RTCs were identified and prioritized in this study by experts from various organizations, including police, vehicle engineering, engineering, emergency, and culture and behavior. 145 RQs were identified using the CHNRI method, 20 of which were determined as high priority research inquiries. Experts have emphasized the critical importance of the 20 top RQs in “road safety management” and “road and infrastructure” pillars. Among 145 identified RQs, the most frequently asked questions were in the field of “road safety management”.

A road safety management system is a critical for ensuring the long-term promotion of road safety through cost-effective initiatives [23]. It encompasses institutional accountability, data- driven countermeasures and evidence-based policymaking [24]. Bliss et al (2012) discussed road safety management challenges during the first decade of action for road safety (2011-2020), indicating a high level of sophistication in LMICs [25]. A bibliometric analysis revealed that “road safety management” was one of five topic clusters with a high frequency of occurrence in published documents in the field of road traffic injury research between 1928 and 2018 [26]. On the contrary, most research focuses on developed countries with several conducted in LMICs [27].

Road safety management is a priority area for countries in the WHO's Eastern Mediterranean regional office (EMRO) area, as it was identified as barrier in Kuwait's national traffic and transportation strategy (2011-2019) [28]. In Iran, non-integrated road safety management has been identified as a barrier to road safety promotion [17,29]. The high frequency of RQs in this study's pillar, “road safety management” indicated that experts were well versed in the subject. Developing key-responses to RQs in this field will provide valuable information for institutional management function such as accountability, financial management, legislation, coordination, promotion and knowledge transfer [30]. Lead Agency was one of the most important road safety [31]. Lead Agency is critical issue in Iran which causes a magnitude of problems which it comes to managing RTCs. National experts examined the challenges associated with road traffic management in the 2019 in the project of National Road Safety Plan (NRSP). national experts [17]. Investigations into leading agencies will strengthen national organizational capacity for effective policymaking and management [17,31]. Budgeting for road safety, inter-sectoral collaboration, public participation, knowledge transfer, legislation and law enforcement and information systems are all areas that could be explored under the “road safety management” pillar.

“Road and infrastructure” was the second pillar with the most RQs suggested by experts. A safer road and infrastructure have an intrinsic effect on the crash risk because they shape how users perceive their environment [[32]. According to one study, a lack of road network and infrastructure, particularly in LMICs, has increased burden of road traffic injuries [[33]. According to Azami et al (2019), scientific barriers such as a lack of evidence-based practice, insufficient knowledge transfer and benchmarking, and knowledge production are significant impediments to road traffic injury reduction in Iran [34]. Research in this area will contribute to our understanding of road network safety and sustainability, RCTs and RTIs statistics. It is recommended that Iran's Road Safety Commission supervise and fund research in this field for both inner and outer city road networks.

Out of 145 research questions, those pertaining to “road user behavior” were identified as the second priority pillar. Among the top 20, the “Road user behavior” pillar had the fewest proposed RQs. Road user behavior has as a risk factor for RTIs identified [1]. Examining these behaviors and the efficacy of various strategies for modifying them has significant implications for developing public policies regarding safe behaviors.

The frequency of RQs in “safer vehicle” and “post-crash care” pillars is identical. Vehicle safety is a contentious issue in road traffic safety in Iran, as automobile imports are restricted, and there are no comprehensive vehicle safety monitoring systems in place, such as the new car assessment program (NCAP) [35]. Concerning notable advancement in related technologies used in vehicle safety, researching related topics will wield novel findings that will help determine a path forward for vehicle safety promotion in Iran.

The services provided to road traffic accident victims are typically divided into two stages: pre-hospital and in-hospital. The findings of studies indicated that the mortality rate of RCTs was high in Iran, both pre-hospital and at the scene of the crash. For example, in crashes involving pedestrians, more than 31% of pedestrian fatalities occurred on the way to the hospital, and more than 19% occurred in hospitals [36]. It is while; a big percentage of RTC mortality in Iran can be prevented by improving the quality of medical services. Determined RQs address trauma care timeliness, quality and safety; the comprehensiveness of emergency medical services; and the development of registry systems, among others, all of which will require valuable responses to overcome the deficiencies.

The highest score for RPs was observed for “Investigating the effects and strategies of developing intelligent systems to achieve national safety goals “. This priority pertains to both “road safety management” and “infrastructure”. Intelligent systems are widely used to improve road safety. By utilizing intelligent systems road user' and traffic crash partner organizations' access to information can be improved [37].

The second priority of this study “exploring ways to achieve public participation in promoting traffic safety” is related to “road safety management” and “road user behavior.” Utilizing the capabilities of individuals and non-governmental organizations is a critical component of promoting road safety. In the section on vehicle safety strategies of the National road safety plan, which was developed with the widespread participation of numerous experts and specialists in RTCs, a strategy titled “Development of public participation policies to increase demand for safe vehicles” is presented. By involving the public in road safety, we can accomplish the goals of mandating the use of critical safety technologies in vehicles, continuously improving the quality of safety standards, and finally, providing the market with safer vehicles. Additionally, the national road safety plan considered the role of public participation in promoting traffic culture and behavior [17].This study has some limitations. A significant effort was made to include experts from a diverse range of fields related road safety. However, the proposed RQs may be biased due to the absence of experts who agreed to participate in the study. This non-response bias could affect the results.

## CONCLUSIONS

The study's findings indicated that the majority of RQs were “road safety management.” The findings of this study may contribute to the most efficient use of resources in the long-term goal of preventing and controlling road traffic crashes and their related injuries. Considering these RQs as research investment options will advance the status of RTIs at the national level accordance with international objectives. If these research priorities are pursued, and their findings are implemented, we can anticipate a significant reduction in the number of crashes, injuries, and deaths.

## Additional material


Online Supplementary Document
