# Supplementary material for: Setting research priorities to achieve long-term national road safety goals in Iran
Source: J Glob Health. 2022 Apr 2;12:09002. doi: 10.7189/jogh.12.09002 (PMC8974318; doi:10.7189/jogh.12.09002)
Supplement: Online Supplementary Document [file jogh-12-09002-s001.pdf]

Supplementary

Table S1. Characteristic of experts participated in the study

Experts field	Work experience (year)	Working sector	Interests
Road Traffic Management	32	Traffic Police	Road Traffic Management
Injury Prevention and Safety Promotion	29	University/ Faculty Member	Safety promotion, Injury prevention, Road Traffic Injury prevention
Strategic Management/ Traffic Management	33	Traffic Police	Transportation,<br>Strategic Management,
Civil engineering	33	University/ Faculty Member	Road safety
Health policy	7	University/ Faculty Member	Health policy,<br>Health system research
Health in disaster and emergencies	26	University/ Faculty Member	Health in disaster, Injury
Transportation engineering	35	University/ Faculty Member	Traffic safety,<br>Traffic operation and management,<br>Traffic flow modeling
Transportation engineering	10	University/ Faculty Member	Road traffic analysis, Road safety audit
Energy and sustainable development/ Architecture	18	University/ Faculty Member	Sustainable development, Sustainable city, Intelligent transportation system, Benchmarking
Injury Epidemiology	30	University/ Faculty Member	Road traffic injury epidemiology
Psychiatry	26	University/ Faculty Member	Traffic and mental health
Pharmaceutical Economics and management	15	University/ Faculty Member	Road traffic economy
Mechanic engineering	9	University/ Faculty Member	Autonomous vehicles, Advanced driving Assistance System,<br>Vehicle Braking system

Experts field	Work experience (year)	Working sector	Interests
Biostatistics	15	University/ Faculty Member	Road Traffic accident
Criminal Law and Criminology / Social adjustment for road traffic accident prevention	34	Traffic police	Criminal Law and Criminology ,<br>Traffic culture
Health education and health promotion	8	University/ Faculty Member	Lifestyle, Non-communicable disease prevention
Emergency medicine	12	University/ Faculty Member	Emergency,<br>Health in disaster
Traffic / urban planning	19	Traffic police	Child pedestrian safety, Motorcyclists' safety
Aerospace engineering	8	University/ Faculty Member	Driving simulator,<br>Advanced driver-assistance systems,<br>Vehicle safety
Trauma biomechanics	9	University/ Faculty Member	Trauma biomechanics, Impact biomechanics,<br>Traumatic brain injury
Health management	4	University/ Faculty Member	Traffic injury,<br>Road safety management
Crisis strategic management	30	University/ Faculty Member	Crisis and strategic management,<br>Traffic management,<br>Traffic culture
Civil engineering	20	University/ Faculty Member	Civil Engineering,<br>Intelligent transportation system
Traffic management	30	Traffic police	Traffic management

Table S2. All 145 RQs for Road Traffic Safety in Iran

Item Number	Proposed RQ*	Feasibility (Score)	Impact on health (Score)	Impact on economy (Score)	Capacity building (Score)	Equity (Score)	RPS† (Score)	wRSP ‡ (Score)	AEA § (Score)	Road safety pillar (Code)
1	Investigating the effects and strategies of developing intelligent systems to achieve safety goals in the country	66	70	65	69	57	65.4	55.8	19	1, 2
2	Exploring ways to achieve public participation in promoting traffic safety	64	70	66	63	60	64.6	55.2	19	1, 4
3	The investigation of methods for utilizing the experience and knowledge of experts and educated individual in the field of traffic safety	70	65	59	68	60	64.4	54.7	19	1
4	The study and compilation of significant RTC analysis programs by specialized crash analysis teams in conjunction with an in-depth examination of the crash scene in order to determine the causes of crashes and their severity	70	68	57	65	56	63.2	53.7	17	1, 5
5	Study to assess and continuously improve the quality of RTCs-related health care services	66	69	60	58	63	63.2	53.9	17	5
6	Conducting pre and post intervention studies to ascertain the efficacy of road safety interventions	72	65	66	63	49	63.0	53.7	17	1
7	Evaluation of low-cost methods for improving road safety	70	67	68	52	58	63.0	53.9	18	1
8	Examining the possibility of amending road traffic-related legislation in order to improve road traffic safety	71	67	61	58	57	62.8	53.5	17	1
9	Reforming macro-level traffic safety policies	66	61	57	69	57	62.0	51.9	17	1

Item Number	Proposed RQ*	Feasibility (Score)	Impact on health (Score)	Impact on economy (Score)	Capacity building (Score)	Equity (Score)	RPS† (Score)	wRSP ‡ (Score)	AEA § (Score)	Road safety pillar (Code)
10	Study of the role of enforcing police regulations using modern and intelligent tools promote road traffic safety	63	64	68	58	55	61.6	52.7	18	1
11	Study of reviewing and developing a comprehensive RTCs registration system and analyzing indicators	70	63	57	60	57	61.4	49.1	16	1
12	Study of the challenges related to improving the safety of interurban and rural roads	68	62	58	64	54	61.2	52.0	17	2
13	Preliminary feasibility studies for establishing a vehicle rating and reporting system (accreditation) with the assistance of vehicle insurance companies	64	65	56	57	63	61.0	52.0	17	3
14	Study of the Lead Agency in road traffic crashes in Iran	65	66	63	57	54	61.0	52.1	16	1
15	Reviewing the road traffic rules related to driving violations in order to promote road safety and driving culture	65	60	58	64	58	61.0	47.5	17	1, 4
16	What strategies are in place to develop continuous training of safe traffic behaviors for professional drivers (public transport drivers including buses, trucks, vans, and taxis)?	60	66	61	64	53	60.8	51.9	16	1, 4
17	Study of mechanisms for boosting knowledge and industry exchange in the field of traffic safety	68	66	58	58	54	60.8	42.4	17	1
18	Study the possibility of an intelligent system for detecting driver fatigue and drowsiness	62	64	57	60	60	60.6	51.6	17	2, 4
19	Study of establishing a connection between national intelligent systems such as the meteorological system, the	65	63	61	62	52	60.6	51.6	15	2

Item Number	Proposed RQ*	Feasibility (Score)	Impact on health (Score)	Impact on economy (Score)	Capacity building (Score)	Equity (Score)	RPS† (Score)	wRSP ‡ (Score)	AEA § (Score)	Road safety pillar (Code)
	police system, and the breakdown monitoring system on improving road traffic safety									
20	Methodological study the process of attracting, providing, and allocating financial resources for road traffic infrastructure development in order to achieve sustainable development in the field of road traffic safety	65	70	58	59	51	60.6	51.6	17	2
21	The study of the relationship between intelligent national systems such as meteorological, police, and crash monitoring systems, as well as their effect on improving road safety	62	62	62	59	56	60.2	51.7	17	2
22	The study of methods for attracting, providing, and allocating financial resources for the creation and development of transportation infrastructure in order to achieve the road safety sustainability goals	64	55	66	55	60	60.0	51.3	16	1, 2
23	The examination of operational strategies aimed at reducing the demand for intra-city travel	67	64	65	55	48	59.8	51.1	17	1, 2
24	Examination of effective methods for preventing dangerous pavement failures	61	63	62	54	57	59.4	52.6	17	2
25	Economic analysis of the costs of road crashes caused by unsafe vehicles in comparison to the liberalization of high-safety vehicles	62	63	67	53	50	59.0	50.6	16	3
26	Study the possibility of psychological tests administered at the start of the certification process for all drivers, or the very least for professional drivers (buses, trucks, suburban taxis, and others), and repeated every 5 years	66	68	53	60	48	59.0	50.1	16	4
27	Aggregation of data on road traffic collisions from all relevant organizations	63	65	55	59	52	58.8	50.1	16	1

Item Number	Proposed RQ*	Feasibility (Score)	Impact on health (Score)	Impact on economy (Score)	Capacity building (Score)	Equity (Score)	RPS† (Score)	wRSP ‡ (Score)	AEA § (Score)	Road safety pillar (Code)
28	Investigation of solutions designed to improve motorcycle safety	60	63	57	53	60	58.6	50.1	15	3
29	The examination of the challenges and solutions inherent in the development and effective operation of intelligent road traffic management systems	64	61	58	60	50	58.6	49.9	15	1
30	The investigation of strategies for enticing the public to participate in promoting safe driving behaviors for various groups of road users	60	63	57	60	52	58.4	49.8	16	1, 4
31	Examination of the performance of road safety audits during the road construction phase	61	60	61	57	53	58.4	49.9	17	2
32	The study of the roads attributable risk in road traffic collisions and related injuries	64	62	59	54	52	58.2	49.7	15	1
33	Control and revision of accident registration forms, as well as the elimination of existing deficiencies and database development strategies	70	65	53	63	39	58.0	49.2	15	1
34	The investigation of strategies for improving motorcyclists' safety behavior concerning helmet use	63	67	50	53	57	58.0	49.3	16	1, 4
35	In collaboration with experts in the field of road safety, develop a comprehensive safety plan for the development of intelligent transportation technologies and ITS	63	63	59	58	47	58.0	46.4	15	1
36	The effect of laws on high-risk drivers, particularly public transportation drivers, in terms of promoting road safety	59	68	57	48	57	57.8	49.5	16	1
37	The study of families with children's knowledge, attitudes, and behaviors regarding the use of child seats	64	69	54	52	49	57.6	49.1	14	4
38	Examination of the treatment measures administered to injured patients (and deceased) in medical centers	64	66	52	51	55	57.6	49.1	15	5
39	The study of the growth of green transportation in cities to promote traffic safety	62	65	56	56	49	57.6	45.7	15	1, 2

Item Number	Proposed RQ*	Feasibility (Score)	Impact on health (Score)	Impact on economy (Score)	Capacity building (Score)	Equity (Score)	RPS† (Score)	wRSP ‡ (Score)	AEA § (Score)	Road safety pillar (Code)
40	The development and evaluation of in-vehicle interaction systems to reduce passive fatigue under consistent driving conditions	61	67	55	54	49	57.2	48.8	13	3
41	Assessment of the economic cost of road traffic crashes on a national scale	67	57	59	51	51	57.0	48.6	14	1
42	Investigate the road safety challenges and solutions faced by elderly road users (pedestrians, passengers, and drivers)	65	67	46	52	55	57.0	46.1	14	4
43	A comprehensive study of strategies for enforcing traffic laws and effective interventions for traffic violations	64	54	55	57	54	56.8	43.5	15	1, 4
44	A study of how to manage police deployment on the roads in response to the dynamic changes in accident hotspots	63	68	54	55	44	56.8	48.2	14	1, 2
45	Investigate and support the growth and development of knowledge-based businesses and STARTUPS in the field of road safety	65	56	56	59	47	56.6	48.2	14	1, 2
46	The study of developing a national decision-support system for road safety, including a national registry system and a system for disseminating scientific information	59	58	57	57	50	56.2	47.9	15	1
47	A study of vehicle attributable risks in road traffic collisions and related injuries	64	60	57	52	48	56.2	47.9	14	1, 5
48	A study of the critical roles of transportation policymakers	59	56	54	57	54	56.0	47.7	15	1
49	Study the effect of educational content on a driver's safe behavior following the issuance of a driver's license	63	64	47	54	52	56.0	47.5	14	4
50	Developing preventive monitoring systems for driver fatigue-related behaviors	60	65	57	51	46	55.8	47.7	14	4

Item Number	Proposed RQ*	Feasibility (Score)	Impact on health (Score)	Impact on economy (Score)	Capacity building (Score)	Equity (Score)	RPS† (Score)	wRSP ‡ (Score)	AEA § (Score)	Road safety pillar (Code)
51	Examination of the inadequacies in the executive guarantee for carrying out the responsibilities of related organizations in the field of road safety	61	57	53	56	52	55.8	43.6	14	1
52	Cost-effectiveness analysis of interventions promoting safe driving behaviors among road users	59	56	59	53	51	55.6	47.53	13	1, 4
53	A comparative study of a traffic police officer skill training course in comparison to leading countries in road safety	66	55	49	61	46	55.4	49.5	13	1
54	Examining and standardizing the specialized training required of traffic police officers and the Association of Accident Experts	69	55	42	60	50	55.2	46.5	14	1, 5
55	The investigation of strategies for the prevention of driving under the influence (DUI)	62	63	54	52	45	55.2	47.1	13	1, 4
56	Analyze the evolution of the role of non-governmental organizations (NGOs) in promoting road safety	58	55	51	58	52	54.8	46.6	13	1
57	Conduct research into the requirements for institutionalizing safe behaviors in the general public	61	59	52	56	46	54.8	46.6	14	4
58	Study of the experiences with road safety knowledge over the last decade: Identifying strengths and weaknesses	67	57	52	59	39	54.8	46.5	13	1
59	The study of strategies for decommissioning worn-out cars that consume a high level of fossil fuels and emit pollution based on a three-step process	55	60	58	53	48	54.8	36.4	13	3
60	Investigating the potential for renewable energy to be used in electric and hybrid vehicles	57	58	56	55	48	54.8	52.2	13	3
61	Standards that will be required in the future to accomplish the goals associated with agents of safety vehicles and roads	58	57	56	55	47	54.6	46.6	14	1, 4

Item Number	Proposed RQ*	Feasibility (Score)	Impact on health (Score)	Impact on economy (Score)	Capacity building (Score)	Equity (Score)	RPS† (Score)	wRSP ‡ (Score)	AEA § (Score)	Road safety pillar (Code)
62	A comparative study of methods for promoting and enforcing safe driving behaviors among road users	58	58	52	54	51	54.6	45.4	12	4
63	Investigate the feasibility of developing and communicating comprehensive road traffic laws in collaboration with stakeholders	59	61	50	55	47	54.4	37.6	13	1
64	The study of the difficulties faced by disabled road users and the development of a solution to improve road safety	63	61	45	50	53	54.4	44.7	13	4
65	Study the role of the government and policymakers in achieving the goal of reducing road traffic fatalities by 10% annually	56	60	56	51	48	54.2	46.3	14	1, 5
66	The study of strategies for enhancing vehicle and road safety in the face of sanctions and the people's and government's economic conditions	59	57	56	54	45	54.2	46.2	13	1, 4
67	Investigate emerging technologies in the field of road safety and develop strategies for implementing them in Iran	57	58	57	56	43	54.2	46.3	12	1, 2
68	The study of the difficulties associated with driver safety training programs and the formulation of recommendations for enhancing driver safety behavior	61	56	50	56	48	54.2	46.1	13	4
69	Examination of strategies for effectively enforcing the legal provisions governing traffic violations, particularly those governing how the proceeds of violations are spent	57	52	59	50	53	54.2	46.9	14	1, 4
70	Examining strategies for promoting national broadcasting's role in promoting road safety	65	57	48	48	52	54.0	48.4	14	1
71	Examine the difficulties and solutions associated with the issuance of a driver's license	62	58	50	52	47	53.8	45.7	13	1
72	Investigation of strategies for reducing motorcycle trips and establishing the types and specifications of motorcycles that may be used in traffic	56	64	51	45	52	53.6	45.8	12	1, 4

Item Number	Proposed RQ*	Feasibility (Score)	Impact on health (Score)	Impact on economy (Score)	Capacity building (Score)	Equity (Score)	RPS† (Score)	wRSP ‡ (Score)	AEA § (Score)	Road safety pillar (Code)
73	Assessment of the cost-benefit ratio of road safety interventions	64	48	65	44	47	53.6	45.9	13	1
74	The study of the factors that contribute to lawlessness in Iranian society, particularly while driving	59	62	52	49	46	53.6	45.7	13	1, 4
75	Study the effect of a lack of appropriately trained trauma medical personnel on the road traffic accident fatality rate	56	60	52	52	48	53.6	45.7	14	1, 5
76	Evaluation of various strategies for addressing driver fatigue	56	62	52	53	45	53.6	45.7	14	4
77	Study the effectiveness of education for children and adolescents between pre-school and high school in promoting safe road user behavior	60	59	46	53	50	53.6	46.8	15	4
78	Study of determining the level of recognition of high-risk situations in drivers	60	65	51	48	43	53.4	46.3	13	4
79	Study the effect of simulator training on the behaviors of novice drivers	66	55	49	54	42	53.2	45.1	13	4
80	Study local approaches that are both effective and feasible for reducing traffic accidents in the country	60	60	53	47	46	53.2	45.4	13	1
81	Examination of the evolution of smart-green transportation infrastructure	58	55	54	51	48	53.2	42.2	13	2
82	Study the development of an intelligent detection system and an obstacle warning system for vehicles	62	64	49	49	41	53.0	45.1	13	3
83	Study the promotion of comprehensive data collection sources for road traffic accidents and development of a validation system for verifying the collected data	63	57	47	54	44	53.0	44.9	11	1
84	Study of establishing national and provincial research networks in collaboration with stakeholders in order to reduce traffic accidents	55	58	49	56	47	53.0	45.1	12	1

Item Number	Proposed RQ*	Feasibility (Score)	Impact on health (Score)	Impact on economy (Score)	Capacity building (Score)	Equity (Score)	RPS† (Score)	wRSP ‡ (Score)	AEA § (Score)	Road safety pillar (Code)
85	The examination of the effect of an individual's or society's economy on reducing road traffic injuries through the provision of safer vehicles	52	58	57	49	49	53.0	45.4	11	3
86	Examine economic factors affecting road traffic injuries and fatalities	54	51	58	48	54	53.0	48.3	13	1
87	Study on determining the utilization of intelligent systems in controlling seat belts worn by intercity bus passengers	65	65	47	45	43	53.0	48.4	12	4
88	Study the effect of using a simulator driving system in driving training centers	65	57	51	52	39	52.8	44.8	13	4
89	Study implementation of a road safety performance management system through the use of a GIS online system	62	62	48	50	41	52.6	41.2	13	2
90	Study the role and effectiveness of the Road Safety Commission and the Supreme Council for Traffic Coordination	61	54	49	54	44	52.4	44.5	13	1
91	Examining the effects of dominant law enforcement by the traffic police on motorcyclists' helmet wearing behavior	57	58	51	47	48	52.2	44.5	11	1, 4
92	Euro NCAP assessment of a vehicle's passive safety	59	58	52	48	44	52.2	44.5	14	3
93	Investigation of strategies for increasing motorcycle riders' visibility	59	62	49	46	45	52.2	44.5	12	4
94	Study the development of an integrated traffic culture system to enhance road safety	59	56	50	48	47	52.0	44.3 1	12	4
95	Predicting the effect of income and wealth inequality on the type and quality of services available in road traffic	53	57	54	47	49	52.0	44.5	13	1
96	Study the effect of education, campaigning, marketing, and culture-building interventions on improving road safety	62	55	47	49	47	52.0	45.8	12	1

Item Number	Proposed RQ*	Feasibility (Score)	Impact on health (Score)	Impact on economy (Score)	Capacity building (Score)	Equity (Score)	RPS† (Score)	wRSP ‡ (Score)	AEA § (Score)	Road safety pillar (Code)
97	Establishing a national vision for road safety for the next two decades and developing strategies for achieving related goals	54	58	55	52	40	51.8	44.3	12	1
98	The study of the difficulties associated with organizations' parallel activities regarding traffic safety culture	58	49	54	52	45	51.6	43.9	12	1, 4
99	Examination of strategies for achieving the annual reduction target of 10%	44	58	58	48	49	51.4	44.2	13	1
100	A study of financial incentives for drivers and pedestrians to improve their safety behaviors	58	52	57	46	44	51.4	43.9	11	4
101	Study the economic impact on driving cultures	54	54	57	45	47	51.4	44.1	11	1, 4
102	Study the effect of difficult service conditions on the efficiency of traffic police officers	59	53	47	52	46	51.4	46.9	11	1
103	Examining the effect of traffic sign placement on accident-prone axes of road traffic and determining their role in accident occurrence	63	63	52	40	38	51.2	43.7	12	1
104	Investigate strategies for increasing consumer demand for safer vehicles	51	53	51	52	49	51.2	43.7	14	1, 3
105	Examine the effects of local training programs on improving road safety behavior with the cooperation of all social capacities	50	57	46	53	49	51.0	43.4	14	4
106	The investigation of long-term strategies for avoiding or mitigating the effects of change management in government	54	55	51	50	45	51.0	45.4	11	1
107	Study the development of intelligent in-vehicle systems to enhance road safety in the short term	48	56	55	54	40	50.6	43.3	13	3
108	Developing and evaluating systems for modifying and improving the inactive sitting position of the driver in	54	59	50	47	41	50.2	42.8	10	3

Item Number	Proposed RQ*	Feasibility (Score)	Impact on health (Score)	Impact on economy (Score)	Capacity building (Score)	Equity (Score)	RPS† (Score)	wRSP ‡ (Score)	AEA § (Score)	Road safety pillar (Code)
	order to alleviate physical and mental fatigue									
109	Examining strategies for enhancing national technical expertise in the field of road safety	54	51	47	54	44	50.0	42.5	13	1
110	Examination of the psychological service requirements at the scene of a traffic accident	54	56	42	51	46	49.8	42.3	13	5
111	The study of the factors that contribute to road users' decreased risk perception	58	60	47	46	38	49.8	46.5	11	4
112	Comparing the rate of road traffic collisions between disabled and non-disabled drivers and proposing a preventive measure	56	51	47	43	49	49.2	41.9	11	4, 5
113	Investigate the long-term psychiatric needs of injured patients and propose a solution	59	58	40	43	46	49.2	41.7	11	5
114	Study the development of a systematic process for establishing a car tax system that is based on the technical characteristics of the vehicle	54	47	57	41	47	49.2	41.2	10	1, 3
115	Examine the standardization of advertisement boards on the outskirts of suburban thoroughfares to minimize driver distraction	65	58	49	39	34	49.0	41.7	10	4
116	Study the factors that contribute to the lower quality of some domestically produced vehicles and the development of a solution for enhancing vehicle safety	54	55	51	40	45	49.0	41.9	11	3
117	Identifying the short- and long-term consequences of road traffic accidents and their outcomes, as well as the most appropriate management solutions	53	54	51	48	39	49.0	41.8	10	1, 5
118	Examination of strategies for incorporating road safety components into all national programs and policies	50	54	50	50	40	48.8	41.7	12	1

Item Number	Proposed RQ*	Feasibility (Score)	Impact on health (Score)	Impact on economy (Score)	Capacity building (Score)	Equity (Score)	RPS† (Score)	wRSP ‡ (Score)	AEA § (Score)	Road safety pillar (Code)
119	Study the effect of government and budget change management on the optimal use of forgoing roadside strategies	51	54	53	46	39	48.6	41.6	13	1, 2
120	What are the direct and indirect effects on road traffic injuries of social events such as the COVID-19 pandemic and global warming?	53	52	50	46	42	48.6	41.5	10	1, 5
121	Study the effect of social determinants on drivers' risk perception	61	59	41	41	41	48.6	41.1	11	4
122	Study the development of Electronic Stability Program (ESP) technology for vehicle production	56	60	44	40	42	48.4	41.3	14	3
123	Study the development of parking and rest areas following road safety standards	54	53	47	46	42	48.4	41.2	13	2
124	Study road brightness intensity of intercity roads and establishing an online GIS registration system	53	56	51	44	37	48.2	40.1	9	2
125	Investigate the effect of economic determinants on vehicle drivers' risk perception	52	51	52	43	42	48.0	37.7	10	4
126	Investigate the public's knowledge, attitude, and behavior regarding vehicle safety	52	58	47	42	39	47.6	40.6	11	4
127	Developing the national Road Safety Performance (RSP)	54	49	45	45	45	47.6	40.52	11	1, 2
128	The study of drivers' knowledge, attitudes, and practices regarding child safety seats	52	58	46	42	37	47.0	40.1	11	4
129	The study of strategies for increasing motorcycle riders' motivation to wear high-visibility clothing	56	60	40	40	37	46.6	39.6	10	4
130	Investigate strategies for utilizing a family physician to help prevent road traffic accidents and injuries	46	51	41	49	44	46.2	39.3	10	1, 5

Item Number	Proposed RQ*	Feasibility (Score)	Impact on health (Score)	Impact on economy (Score)	Capacity building (Score)	Equity (Score)	RPS† (Score)	wRSP ‡ (Score)	AEA § (Score)	Road safety pillar (Code)
131	Determining the most effective educational and media interventions for drivers to internalize road safety behaviors	49	52	41	50	39	46.2	39.2	10	4
132	Calculation of the coefficients of correction for national accidents	52	52	42	45	38	45.8	38.9	9	1
133	The study of drivers' knowledge, attitudes, and practices regarding passenger safety	55	54	40	44	35	45.6	38.7	10	4
134	Establishing the law enforcement officer's role in pedestrian road safety behavior	52	54	42	44	32	44.8	38.1	8	1
135	Study the definition and criterion for road traffic fatalities	55	43	38	44	43	44.6	45.5	9	1, 5
136	Conduct a risk assessment of related organizations' exposure to human error	45	46	47	46	37	44.2	37.7	10	1, 4
137	The investigation of strategies for establishing organizational coordination among stakeholders in road safety	51	46	43	42	39	44.2	44.2	9	1
138	Investigate solutions for enhancing road safety standards from the community's perspective	48	45	43	44	38	43.6	37.1	8	1
139	Study the road safety requirements for target groups.	46	46	40	37	47	43.2	36.8	10	1, 4
140	Investigate the belief characteristics that influence drivers' risk perception of accidents	51	50	40	39	34	42.8	31.2	7	4
141	Evaluate the effectiveness of displacement and camouflage strategies for speed camera installation	58	44	44	38	30	42.8	36.4	9	1, 2
142	Examining the causes of fatigue and the risks associated with EMS personnel	49	52	34	38	36	41.8	35.5	9	5

Item Number	Proposed RQ*	Feasibility (Score)	Impact on health (Score)	Impact on economy (Score)	Capacity building (Score)	Equity (Score)	RPS† (Score)	wRSP ‡ (Score)	AEA § (Score)	Road safety pillar (Code)
143	Evaluating the cost-effectiveness of developing a national NCAP for safe vehicles	41	41	38	38	33	38.2	32.6	7	3
144	Study the effect of the COVID-19 epidemic on aggressive driving	45	44	35	35	30	37.8	32.1	5	4
145	Investigate the effect of adopted policies in transportation and traffic policies on risk perception	41	39	37	36	30	36.6	45.5	6	1, 4

†RPS –Research Priority Score; ‡wRPS – Weighted RPS; §AEA – Average Expert Agreement
